# A Novel Vision Sensing System for Tomato Quality Detection

**DOI:** 10.1155/2014/184894

**Published:** 2014-09-03

**Authors:** Satyam Srivastava, Sachin Boyat, Shashikant Sadistap

**Affiliations:** ^1^Advanced Electronics Systems, ACSIR, CSIR-CEERI, Pilani, Jhunjhunu, Rajasthan 333031, India; ^2^Karnataka State Open University (KSOU), Jhunjhunu, Rajasthan 333031, India; ^3^Agri-Electronics Group, CSIR-CEERI, Pilani, Jhunjhunu, Rajasthan 333031, India

## Abstract

Producing tomato is a daunting task as the crop of tomato is exposed to attacks from various microorganisms. The symptoms of the attacks are usually changed in color, bacterial spots, special kind of specks, and sunken areas with concentric rings having different colors on the tomato outer surface. This paper addresses a vision sensing based system for tomato quality inspection. A novel approach has been developed for tomato fruit detection and disease detection. Developed system consists of USB based camera module having 12.0 megapixel interfaced with ARM-9 processor. Zigbee module has been interfaced with developed system for wireless transmission from host system to PC based server for further processing. Algorithm development consists of three major steps, preprocessing steps like noise rejection, segmentation and scaling, classification and recognition, and automatic disease detection and classification. Tomato samples have been collected from local market and data acquisition has been performed for data base preparation and various processing steps. Developed system can detect as well as classify the various diseases in tomato samples. Various pattern recognition and soft computing techniques have been implemented for data analysis as well as different parameters prediction like shelf life of the tomato, quality index based on disease detection and classification, freshness detection, maturity index detection, and different suggestions for detected diseases. Results are validated with aroma sensing technique using commercial Alpha Mos 3000 system. Accuracy has been calculated from extracted results, which is around 92%.

## 1. Introduction

**Figure 1:**
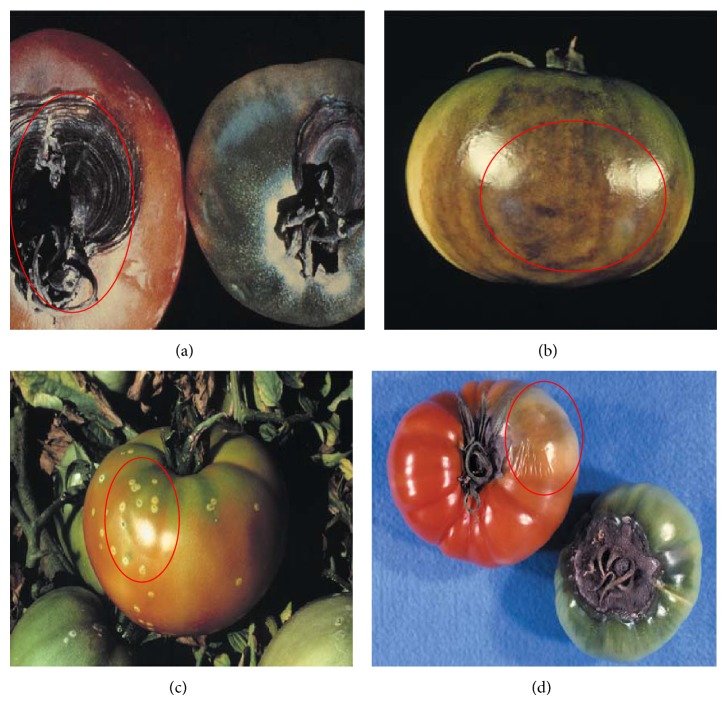
Different diseases effect on the tomato fruit: (a) early blight, (b) late blight, (c) bacterial canker, and (d) gray mold.

The increased awareness and sophistication of consumers have created the expectation for improved quality in consumer food products. This has increased the need of advance quality monitoring systems for quality detection and early warning for different type food samples. Quality itself is defined as the sum of all those attributes which can lead to the production of products acceptable to the consumer when they are combined. The basic quality assessment is often subjective with large number of attributes such as appearance, smell, texture, and flavor, frequently examined by the consumers [1]. Tomato is one of the most consuming fruit samples after potatoes. Tomato is also easily available and cheap fruit for analysis purpose. Tomato is also included in the very major horticulture commodities [2]. Sometimes its demand increases at very high level in market due to low production or damage due to different disease attacks while as supply is limited [3]. Therefore there is an essential need of an advance system for detection of shelf life as well as also provides early warning from various diseases attacks [4]. Quality and productivity control is a big issue in tomato crop due existence of large number of diseases. Most of diseases affect the outer area of the tomato flesh by some color change or different spots. In general there are two types of factors which can affect tomato fruit: living (biotic) and nonliving (abiotic) agents. Different insects, bacteria, fungi, and viruses are the example of living agents. Nonliving agents includes various environment effects such as rapid temperature change, excess moisture, insufficient nutrients, poor soil pH and high humidity conditions [5]. In this kind of scenario vision sensing technique is one of the best as well as appropriate choice to avoid such kind of conditions [6]. Vision sensing technique is very powerful tool for such kind of application with efficient implementation. Color distortion detection, cluster extraction, and entropy calculation are some of very useful techniques used in developed system for prediction of different parameters for tomato fruit [7]. There are large numbers of diseases exists for tomato crop; some of collected samples of different disease attacks are presented in Figure 1. It can easily be figured out that all four diseases affected the outer flesh area of tomato like in Figure 1(a); tomato is affected with early blight disease which generates the black color concentric rings and tomato flesh appearance changes to velvety color. Late blight disease (Figure 1(b)) affects generally unripe tomatoes and it generates irregular brown blotches on the tomato outer surface. While as bacterial canker (Figure 1(c)) and gray mold (Figure 1(d)) affects the outer surface of the tomato like in first one tomato infected with white color spots while as later one generates the light gray spots on the outer wall of the tomato.

## 2. Database Preparation

Experiments were conducted on the fifteen tomatoes divided in three batches taken from local market having different maturity levels. Collected tomato samples have been categorized in standard verities of tomatoes (Table 1). Collected samples have been preserved in different environment condition like first batch stored in the sampling chamber with constant temperature and humidity because there is an essential need to analyze the effect of environment conditions on different parameters like ripening rate, early warning, shelf life, and so forth. Based on past research it can be concluded that at temperature 12°C and humidity at 90% ripening rate is optimum [8]. Preliminary study showed that at constant 20°C temperature and 90% humidity, the initial turning red color changed to deep red in the course of 7-8 days, in parallel with the softening process. Second batch has been preserved in open environment at the room temperature and humidity (*T* = 27°C and RH = 20%) for observing the environment effect on tomato quality. Third and last batch have been preserved in refrigerator at 4°C and humidity at less than 10% chilling environment. Some of the images also have been imported from outside for generating the database of some special kind of diseases because of unavailability of such kind of environment conditions.

Database has been prepared for more than ten living and nonliving bacterial agents exist in tomato presented in Table 2.

## 3. Material and Methods

Firmware development consist of two sections: first one is hardware development composed of developed ARM based system with required experimental set up and second one is algorithm development using various algorithms required for sample classification, early warning about disease attack detection and classification.

### 3.1. Hardware Development

**Figure 2:**
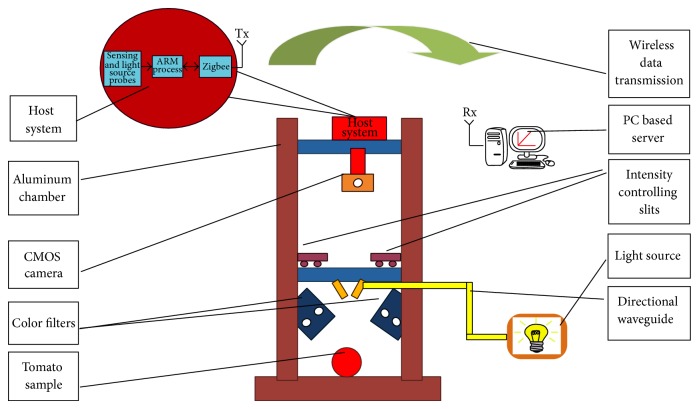
Experimental setup for tomato image acquisition.

An ARM based system consists of USB camera for image acquisition, white light source for providing light, touch screen with qtopia based GUI for controlling purpose, movable slits for optimum light transfer, Zigbee module for wireless image transfer, filters for changing the wavelength of light source, and a PC based server for analysis purpose. The whole setup was installed in black color aluminum chamber to avoid external light as shown in Figure 2. Processor triggers the low power LED based light source interfaced with waveguide before image acquisition. Then image acquisition starts via serial protocol. Processor triggers camera via serial protocol based on the number of frames entered by the user. System also consists of intensity control mechanism performed for efficient light transfer. Movable slits are used for intensity control in the image acquisition process. After every image processor enables different filters for image acquisition in different color space like if processor enables red filter then captured image is in red light domain. This technique is very much useful for color extraction for tomato sample in different color space. Light quality is key point in the image acquisition process for efficient and good quality image acquisition. A low power white light source has been installed in a waveguide for optimum light transfer inside the image acquisition chamber. After image acquisition has been completed then system transfers image on the PC based server using Zigbee module. Server consists of a virtual instrumentation based Matlab based GUI. After image has been received on the PC based server, all image processing related techniques have been Implemented by the system. Different sample detection, classification, and disease detection based techniques execute on the received tomato sample image. Based on the extracted results, server transfers quick subjections to the host systems. All experiments performed in a structured manner for efficient analysis and results for the consumer.

### 3.2. Algorithm Development

Algorithm development consists of sample classification having different subsections like preprocessing, region of interest extraction or segmentation and feature extraction. These subsections extract different parameters related to quality of the tomato sample. Algorithm development phase of the proposed system can be majorly divided into two phases: first one is training and second one is the testing part for the system. Both training and testing require some kind of preprocessing. A higher level of abstraction block diagram for proposed algorithms has been presented in Figure 4.

#### 3.2.1. Preprocessing

**Figure 3:**
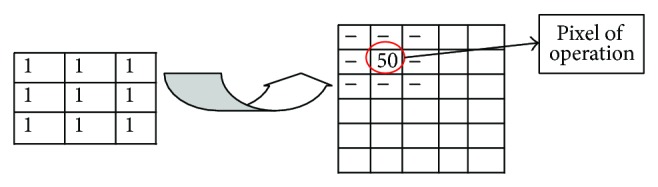
Rank order filter used for noise removal process.

**Figure 4:**
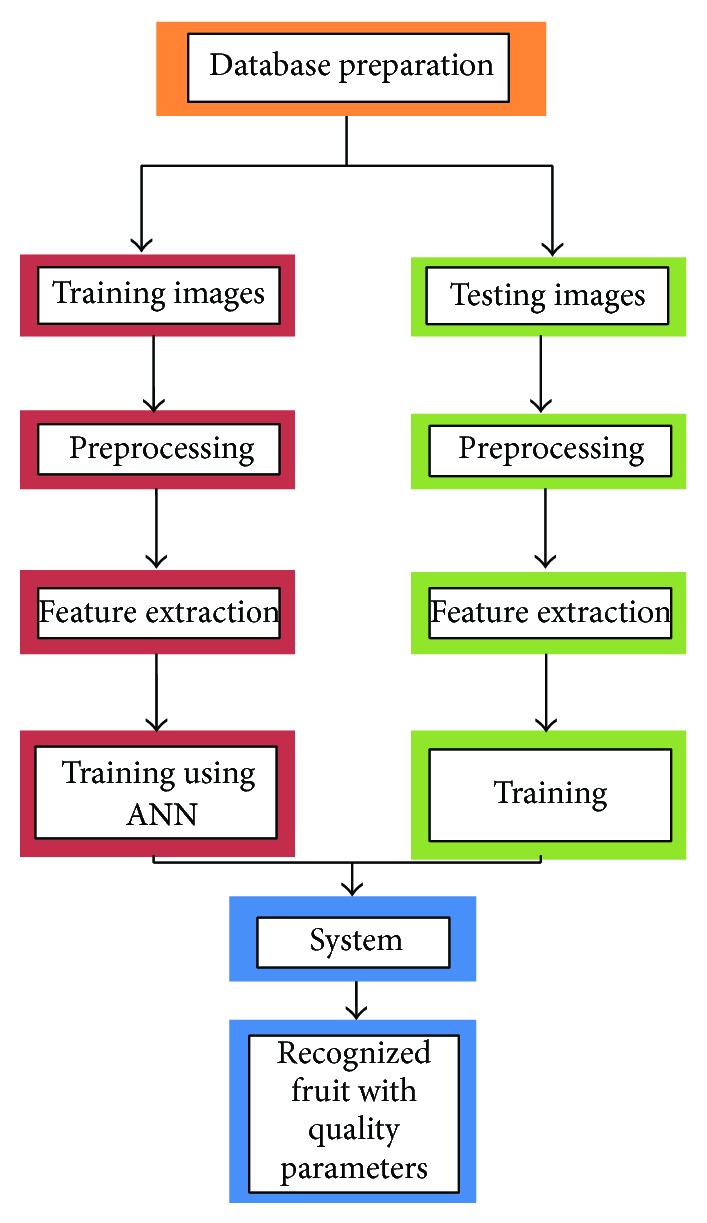
Proposed algorithm for vision sensing system.

Preprocessing section consists of segmentation followed by noise removal subsections. In noise removal rank order filters have been used for cleaning as well as smoothing purpose (see Figure 3). This section removes the noise as well as blur in the captured image. In this subsection, system selects a 3 × 3 mask and moves it on the whole image or it can be understood in more technical way that system performs the convolution of captured image as well as the kernel of the filters.

As rank of the kernel increases, its noise removal ability also increases. All experiments have been performed in a structured environment, So there is very less possibility of noise mixing in captured image. Still due to camera limitations, some of the blur was introduced in the captured image but for such kind of noise removal 3 × 3 kernels are enough. System implements noise removal algorithm from following equation:

(1) $$\begin{array}{c} Y\left(i,j,k\right)=\overset{i=h-1}{\underset{i=3\;}{\sum }}\overset{w-1}{\underset{j=3\;}{\sum }}\overset{b}{\underset{k=1}{\sum }}G\left(i,j,k\right)*\overset{p=i+2}{\underset{p=i-2\;}{\sum }}\overset{q=j+2}{\underset{q=j-2\;}{\sum }}\overset{k}{\underset{r=1}{\sum }}\text{input}\left(p,q,r\right), \end{array}$$

where *G* (*i*, *j*, *k*) is the kernel and input (*p*, *q*, *r*) is the input captured image for noise removal algorithm.

The other preprocessing subsection is segmentation or region of interest extraction. All experiments has been performed in the structured environment inside the chamber, so background subtraction is efficient and optimum for implementing segmentation of region of interest extraction. In this process system captures two successive frames: first one without tomato sample at *t* time and second frame with tomato sample at *t* + 1 time: system subtracts both frames and extracts segmented image. Segmentation process using frame difference method can be explained as follows. It is convenient and effective method for detecting foreground object with stationary background:

(2) $$\begin{array}{c} D\left(x,y,t+1\right)=\overset{i=n}{\underset{i=1\;}{\sum }}\overset{j=n}{\underset{j=1\;}{\sum }}\overset{3}{\underset{k=1}{\sum }}\left(V\left(i,j,t+1\right)-V\left(i,j,t\right)\right). \end{array}$$

#### 3.2.2. Feature Extraction

Feature extraction phase consists of different subsections like color extraction, texture entropy extraction, blots area calculation, and *k*-mean clustering for extraction of different clusters due to different diseases.

(*1) Color Extraction.* Any RGB image is a combination of red, green, and blue colors according to standard color space. So, it is advisable to extract percentage of red, green, and blue components out of all pixels. It helps to detect the maturity level of the tomato. As tomato moves towards maturity level, its color component changes with a variation of maturity level like initially tomato consists of high content of green color, while mature or ripen tomato consists of high content of red color. So using RGB color extraction method we can judge the maturity index of tomato sample. Color extraction is also one of the major key points for detection of tomato sample. This method extracts a range of color from minimum to maximum concentration from captured tomato sample image. Red, Green and Blue color concentration has been extracted using following mathematical transformations:

(3) $$\begin{array}{c} R\left(x,y,z\right)=\text{Input}\left(:,:,2\right)=0,\\ R\left(x,y,z\right)=\text{Input}\left(:,:,3\right)=0,\\ G\left(x,y,z\right)=\text{Input}\left(:,:,1\right)=0,\\ G\left(x,y,z\right)=\text{Input}\left(:,:,3\right)=0,\\ B\left(x,y,z\right)=\text{Input}\left(:,:,1\right)=0,\\ B\left(x,y,z\right)=\text{Input}\left(:,:,2\right)=0. \end{array}$$

(*2) Texture Detection*. Most of the consumers judge quality of the tomato sample based on the texture parameters. Proposed algorithm consists of a texture detection subblock in classification phase. According to traditional information theory, entropy is a measure of uncertainly in a random variable. Entropy is the degree of measure for unusualness presents in a local zone of a captured image. Low entropy images, such as those containing a lot of black spots with very little contrast and large runs of pixels with same or different values. An image is perfectly smooth have very low or zero entropy. This can help for detecting unusualness existing in tomato sample area like a black spot or small bacterial speck. Texture detection is performed on segmented captured image using entropy calculation method. In this method, first system performs grayscale conversion on segmented image. After grayscale conversion, system extracts local entropy of the image using traditional entropy calculation method. After the extraction of local entropy matrix, system calculates the minimum and maximum pixel existing in the local entropy matrix. The mean of the calculated minimum and maximum pixel range from local entropy matrix will act as texture quality coefficient. Calculated texture quality coefficient from local entropy matrix gives approximate idea about tomato texture. Developed system is trained with wide range of texture quality coefficients extracted from different tomato sample images from prepared database:

(4) $$\begin{array}{c} H\left(x,y,z\right)=\overset{i=n}{\underset{i=1\;}{\sum }}\overset{j=n}{\underset{j=1\;}{\sum }}\overset{3}{\underset{k=1}{\sum }}P\left(i,j,k\right)*\log_{2}P\left(i,j,k\right),\\ P\left(x,y,z\right)=\overset{i=n}{\underset{i=1\;}{\sum }}\overset{j=n}{\underset{j=1\;}{\sum }}\overset{3}{\underset{k=1}{\sum }}\frac{n\left(i,j,k\right)}{m^{2}}, \end{array}$$

where size of the segmented image is *m* × *m* and *P*(*x*, *y*, *z*) is the calculated probability index matrix.

(*3) Area Calculation*. Area calculation algorithm is also implemented in the proposed system. Area is also a key feature related to maturity level of the tomato sample. This block takes care of the tomato sample detection as well as classification as well as detection of different diseases for tomato sample. In this process, system first applies binary image conversion on input grayscale image. After binary image conversion, system applies hard thresholding for removal of unnecessary objects presents in captured image like leaves, trees etc. System estimates the area of the object in converted binary image and calculates approximate area of the object. Calculated area is a scalar quantity, which corresponds roughly to the total number of on pixels in the image but might not be exactly the same because different presents of pixels are weighed differently. Basically system estimates the area of all on pixels in the converted binary image by summing the areas of each pixel in the image. The area of an individual pixel is determined by looking at its 2-by-2 neighborhood. There are six patterns, each representing a different area. Each pixel is part of four different 2-by-2 neighborhoods.

**Figure 5:**
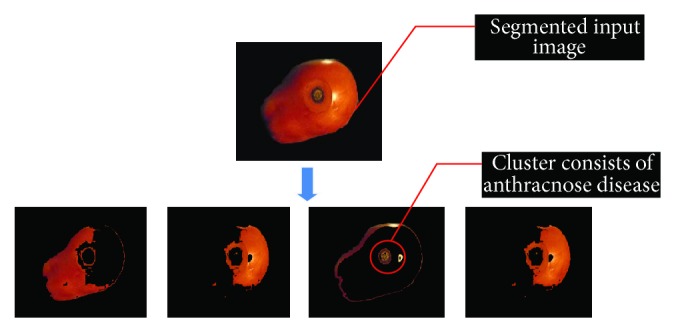
Extracted clusters using proposed algorithm.

- Pattern with zero on pixels (area = 0).
- Patterns with one on pixel (area = 1/4).
- Patterns with two adjacent on pixels (area = 1/2).
- Patterns with two diagonal on pixels (area = 3/4).
- Patterns with three on pixels (area = 7/8).
- Patterns with four on pixels (area = 1).

(*4) Cluster Extraction*. Cluster detection has been implemented for detection as well as classification for different clusters presents in tomato food sample due to different microbial diseases. *k*-mean clustering algorithm has been implemented for cluster extraction and using different feature extraction methods system classifies different clusters (see Figure 5). Basically *k*-mean clustering algorithm classifies the objects (collection of different pixels) into the *k* number of classes based on a set of features. The classification is carried out by minimizing the sum of squares of distances between the data objects and their corresponding clusters. Cluster extraction block first transforms the segmented image from RGB to *L* ^*^ *a* ^*^ *b* color space. Then system classifies different colors using *k*-mean clustering in “*L* ^*^ *a* ^*^ *b*” space. After that system performs labeling process in the segmented image based on the results of previous step. Similar kind of labels generates different clusters presents in processed image. Basically *k*-mean clustering algorithm selects random mean pixel in every cluster presented in processed image and finally extracts the number of clusters presented in image based on the value of *k* that is why *k* should be chosen smartly. Developed system has been initialized by *k* = 4; it means developed system detects only four clusters in every tomato sample. It has been observed that *k* = 4 are able to extract all possible clusters presents in tomato sample. After this whole process system moves mean pixels towards their corresponding clusters based on the calculation of minimum Euclidian distance presented as follows:

(5) $$\begin{array}{c} {\left(\text{E}.\text{d}.\right)}^{2}=\min\left\{\mu_{1}\cdots \mu_{k}\right\}\overset{i=n}{\underset{i=1\;}{\sum }}\overset{j=n}{\underset{j=1}{\sum }}\left|X\left(i,j\right)-\mu^{2}\right|,\\ \mu_{h}=\frac{\sum_{i=1}^{i=n}P\left(i\right)*i}{\sum_{i=1}^{i=n}P\left(i\right)}. \end{array}$$

#### 3.2.3. Supervised Learning and Classification

*Basic* learning algorithms have been implemented for supervised neural networks that are a great use of solving pattern recognition problems. Various pattern recognition algorithms like PCA, LDA, and QDA have been implemented for detection as well as extraction of various patterns generated in different tomato sample frames. Principle component analysis is basically a statistical procedure that uses orthogonal transformation to convert a set of observations of possibly correlated variables in to a set of values of linearly uncorrelated variables called principle components. The number of principle components is less than or equal to the number of original variables. The transformation is defined in such a way that the first principle component has the large possible variance (i.e., accounts for as much of the variability in the data as possible), and each succeeding component in turn has the highest variance possible under the constraint that it is orthogonal to (i.e., uncorrelated with) the preceding components. Developed system performs PCA on segmented tomato samples image and generates different principle components for classification:

(6) $$\begin{array}{c} C\left(x,y\right)=\overset{N}{\underset{q=1}{\sum }}X\left(q,x\right)*X\left(q,y\right),\\ \text{Det}\left(C\left(x,y\right)-\lambda \right)=0, \end{array}$$

and generates eigenvalue *λ*_1_ ⋯ *λ*_*n*_, and eigenvectors *V*_1_ ⋯ *V*_*n*_

(7) $$\begin{array}{c} V=\left[V_{1\;}\cdots V_{n}\right],\\ Y=XV. \end{array}$$

Different discriminant analysis also implemented in developed algorithm for various pattern discriminations. LDA helps to predict the group membership for a number of subjects from a set of predictor variables. The creation variable (grouping variable) is the object of classification. Group variable also acts as a categorical variable, which extracts different data sets collected from segmented tomato sample images. Linear discriminant analysis constructs one or more than one discriminant analysis *D*_*i*_ (linear combinations of the predictor variables *X*_*k*_) such that the different groups differ as much as possible on *D*:

(8) $$\begin{array}{c} D_{i}=b_{0}+\overset{p}{\underset{k=1}{\sum }}b_{k}*X_{k}. \end{array}$$

More precisely, the weights of the discriminate function are calculated in such a way that the ratio (between groups of sample space and within groups of sample space) is as large as possible. First discriminate function *D*_1_ distinguishes first group from groups 2, 3, …, *N* and same as second discriminate function *D*_2_ distinguishes first group from groups 3, …, *N* and same for others. System calculates the optimal weights using a training set consisting of correct classification for a group of datasets of the different tomato samples. After this process, system classifies different data sets using previously calculated discriminate weights to obtain their discriminate scores. Some datasets are too much complex due to close stages of tomato samples, so sometimes LDA is not able to discriminate such kind of data sets. Quadratic discriminant analysis has been implemented to discriminate very much complex data sets of the tomato samples. All the steps in LDA and QDA are same except the classifiers. In LDA linear classifier is used while in QDA second order classifier is used. QDA provides more discrimination capability to the developed system but at the same time it also acquires more memory as well as more processing time. LDA needs to estimate KXP + PXP parameters while QDA needs to estimate KXP + PXPXP parameters. General structure of classifier used in QDA implementation presented as follows:

(9) YQDA=argmink=1,…,K⁡{(x−μk)T∑k=1n(x−μk)−2log⁡⁡(πk)bbbbbbbbbbbbbbbbbbbb+log⁡⁡Z∑k=1n(x−μk)−2log⁡⁡(πk)},Z=1nk∑k=1n(x−μk)∗(x−μk)T.

*k*-nearest neighborhood learning algorithm is also implemented in the proposed algorithm. It provides more accuracy as well as more preciseness in the extracted results. Whenever system has a new point to classify, system find its *k*-nearest neighbors from the training data set. After that system measures the Euclidian distance between pixel and its nearest neighbors from training data set. Distance can be measured by any one of the following distance methods.

- Euclidean distance.
- Murkowski distance.
- Mahalanobis distance.

**Figure 6:**
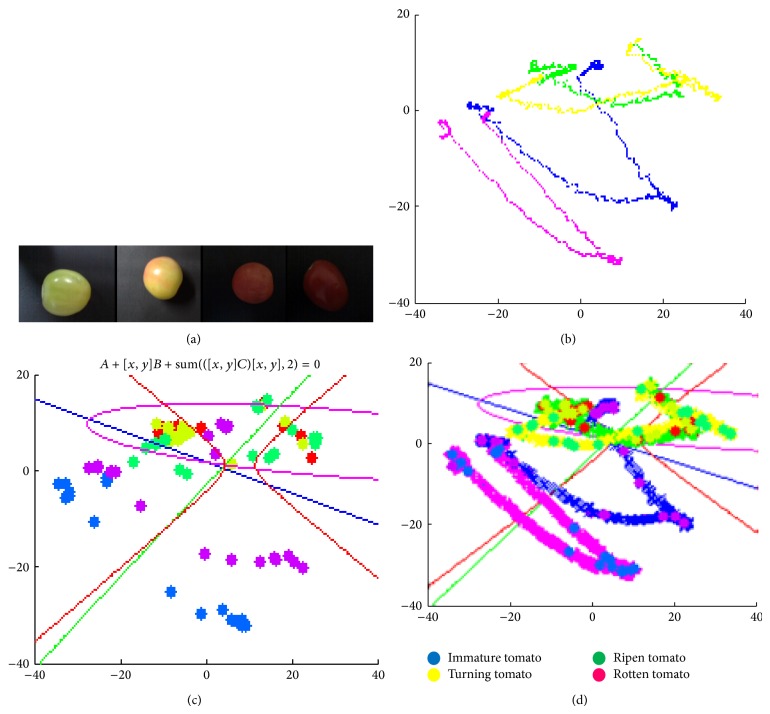
(a) Tomato sample used for experiments having different maturity levels, (b) principle component analysis (PCA), (c) linear discriminant analysis (LDA), and quadratic discriminant analysis (QDA), and (d) *k*-nearest neighborhood analysis.

Implemented algorithm used Euclidian distance for measuring the distance between nearest neighbors from training data set and operation of pixel. PCA, LDA, QDA and KNN have been implemented on four tomato samples having different maturity levels like unripe, ripen, fully ripen and rotten presented in Figure 6.

## 4. Results and Discussions

**Figure 7:**
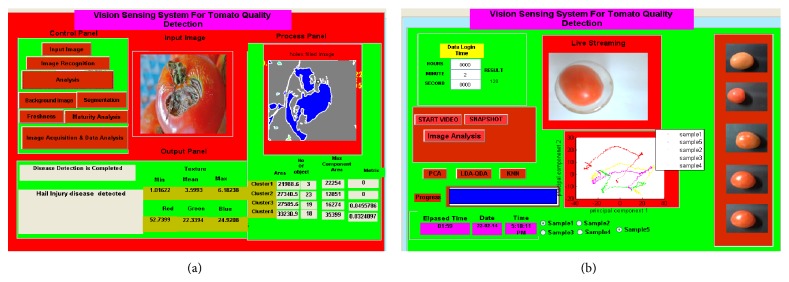
(a) Developed GUI prediction of freshness, maturity level, and disease detection and classification and (b) developed GUI for image acquisition and disease attack prediction.

Developed system has been tested with image of 640 × 480 pixels. Total collected tomato image samples are 100 in that there are 62 healthy tomato samples and 38 tomato image samples are infected from different kind of diseases. Accuracy of the developed system calculated in most of the experiments is around 92%. The method implemented in this research paper is much effective and fast compared to other implemented vision sensing systems. Developed graphical user interface predicts different important parameters related to texture, color, freshness, maturity index, and disease attacks as shown in Figure 7.

**Figure 8:**
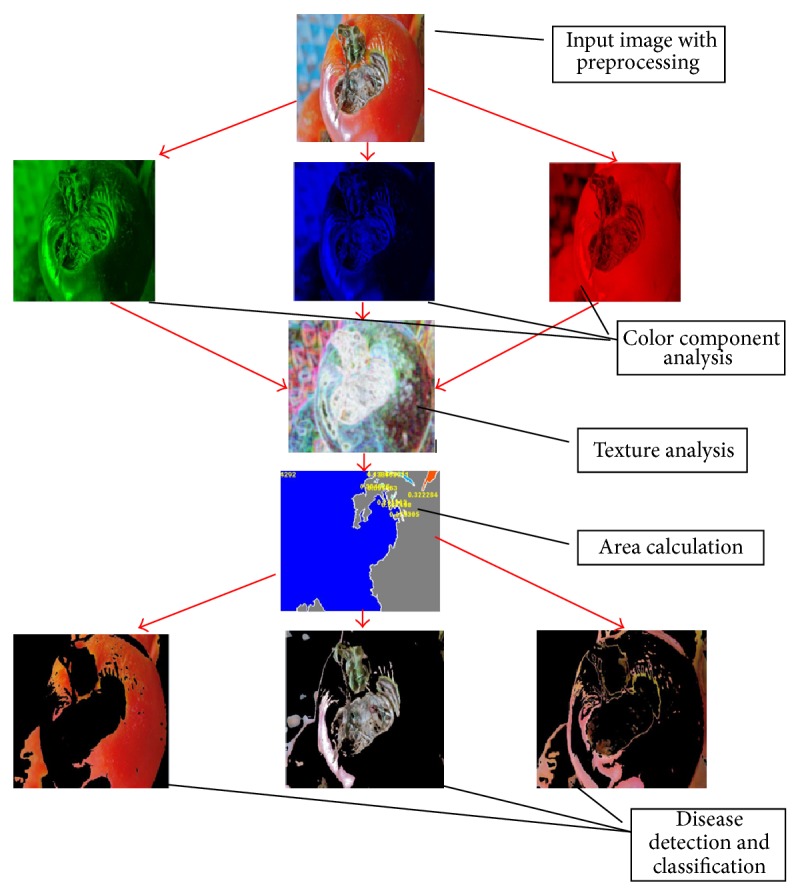
Flow diagram of extracted results for tomato sample.

Developed system is one of the effective as well as fast methods for quality detection of tomato samples using vision sensing technique. The overall results were satisfying and it can be considered as a successful development of system. Presented method is unique in sense of tomato sample detection using different color extraction methods, texture detection methods, and different disease detection and classification methods. A flow of developed algorithm based on extracted results has been presented in Figure 8.

Various parameters have been extracted from developed algorithms such as color index and texture index (entropy), presented in Table 3. It has been observed that color index and entropy calculation is sufficient for maturity index assessment for tomato fruit.

**Figure 9:**
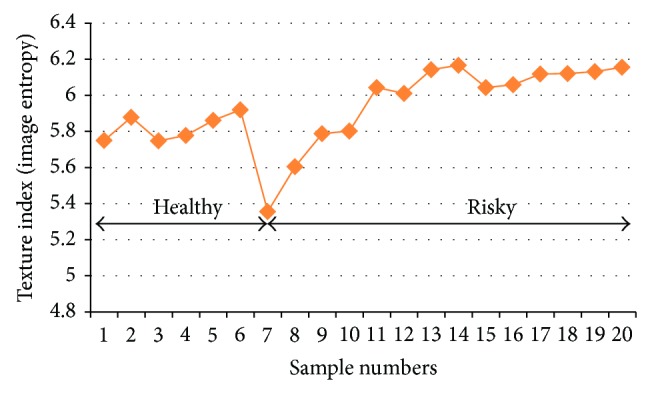
Tomato classification result (healthy and risky).

Infected and healthy tomato samples can segregate based on various extracted parameters such as color index, texture index, and area calculation. It can be observed in Figure 9 that developed system can classify between healthy and infected tomato fruits.

## 5. Validation and Conclusion

Extracted results have been also validated with standard Alpha Mos and ultrasonic based stiffness detection system using aroma and ultrasonic sensing techniques, respectively [10]. It has been observed that extracted results from vision sensing system are very close to Alpha Mos aroma sensing system results. Most of the prediction and results are correct or very close to correct. As a conclusion, this research is strongly recommended to be used for early detection of tomato disease using developed system. Tomato sample images are processed to determine the healthiness of each tomato sample as well as its plant. Developed system is able to detect potential problems in tomato samples. It can predict the attack of disease for tomato sample before the damaging of its crop. Developed system can reduce the use of harmful chemicals on the tomato crop and hence ensure a healthier environment and maybe even lowering the production cost of the maintenance and producing a high quality of tomato.

## Figures and Tables

**Table 1:** Standard varieties of collected tomato samples.

Name of the variety	Characteristics of the variety
Tradiro	Loose tomato with a deep-red fruit color and shiny, a flat round shape, and small size
Clotidle	Cluster tomato with excellent presentation, better flavor than Tradiro, small size and firm
S&G40-290	Loose tomato with a characteristic poor aroma, thin skin, medium size, medium firmness, and less red color then Tradiro

**Table 2:** Database prepared for infected tomato samples for disease detection and classification.

Disease	Symptoms	Collected sample [9]
Early blight	Causing large, sunken areas with concentric rings and a black, velvety appearance	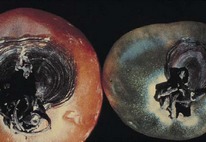

Anthracnose	Symptoms first become visible on ripe or ripening fruit as small, circular, indented spots in the skin. As these spots expand, they develop dark centers or concentric rings of dark specks, which are the spore-producing bodies of the fungus	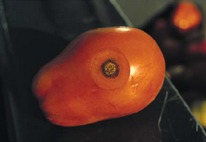

Late blight	Infection of green or ripe fruit produces large, irregularly shaped brown blotches	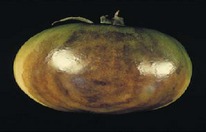

Bacterial spot	Spots on green fruit first appear as black, raised, pimple-like dots surrounded by water-soaked areas. As the spots enlarge to 1/4 to 1/2 inch, they become gray-brown and scabby with sunken, pitted centers	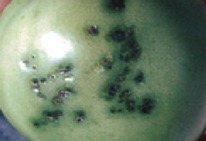

Bacterial speck	Specks that develop on young green fruit are slightly raised, 1/32 to 1/16 inch in diameter, and have well-defined margins	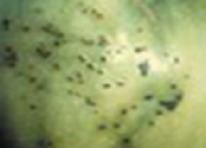

Bacterial canker	White and slightly raised at first, then raised, dark-colored centers with white halos 1/16 to 1/8 inch in diameter. These spots are sometimes termed “bird's eye” lesions	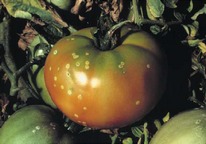

Tomato spotted wilt virus (TSWV)	TSWV causes distinctive yellow ring spots on mature fruit	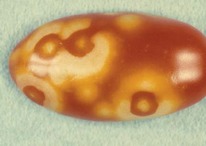

Gray mold	This disease is characterized by a light-gray fuzzy growth that appears on stems and leaves. Soft rot of the stem end of the fruit can also occur	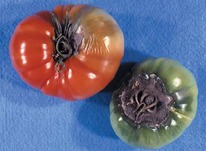

Blossom end rot	It first appears as a sunken, brownish black spot 1/2 to 1 inch in diameter on the blossom end of the fruit. These spots may gradually increase in size	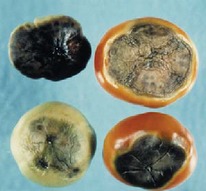

Fruit cracking	Two types of cracks may develop on tomato fruit. Radial growth cracks radiate from the stem, and concentric cracks encircle the fruit, usually on the shoulders	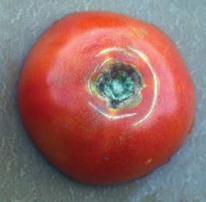

Cat faced fruit	Cat facing is a term used to describe misshapen fruit with irregular bulges at the blossom end and bands of leathery scar tissue	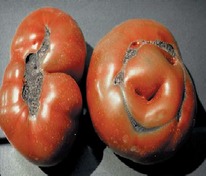

Sunscald	Sunscald occurs on green tomato fruit exposed to the sun. The initial symptom is a whitish, shiny area that appears blistered	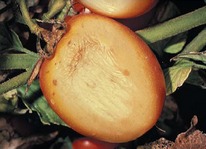

Blotchy ripening	Absence of normal red pigment on localized areas of the fruit. These areas appear as yellow or gray-green patches tomato fruit. When these fruits are sliced open, brown discoloration is often apparent	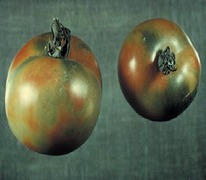

Target spot	Dark brown sunken spots enlarge with cracked centres	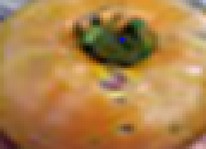

Cloudy spot	Yellow to whitish spots of irregular size; white spongy tissue extends into flesh	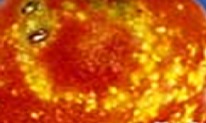

**Table 3:** Preliminary extracted parameters for maturity index assessment.

Sample	*R*	*G*	*B*	Minimum entropy	Maximum entropy	Maturity index
S1	52.3184	23.0222	24.6594	0.448968	5.75004	m
S2	64.398	25.7232	9.87883	0	9.87883	m
S3	85.061	8.09005	6.84894	0	5.74726	m
S4	42.2176	23.0523	34.7301	1.56485	5.778	m
S5	16.6515	39.02	44.3285	1.17164	5.86139	m
S6	37.7259	35.789	26.4851	0.262549	5.9201	u
S7	40.2156	40.9174	18.8671	0	5.35498	u
S8	40.6304	40.5842	18.7854	0	5.60516	u
S9	41.3539	4.3142	18.3318	0	5.78732	u
S10	35.6585	34.7104	29.6311	0	5.80269	u
S11	34.4746	35.456	28.1198	1.51206	6.04355	r
S12	52.2635	17.3957	26.3408	0.323832	5.70998	r
S13	94.6979	3.97336	1.3287	0	6.14232	r
S14	60.5857	36.127	3.30156	0	6.16701	r
S15	31.0084	43.9328	25.0587	0	6.04355	r
S16	57.4464	28.3133	14.2403	0	6.05893	r
S17	43.1682	36.272	20.5598	0	6.11763	r
